# A prospective randomized controlled trial to determine the safety and efficacy of extracorporeal shock waves therapy for primary prevention of subclinical cardiotoxicity in breast cancer patients without a cardiovascular risk treated with doxorubicin

**DOI:** 10.3389/fcvm.2024.1324203

**Published:** 2024-02-07

**Authors:** Shinjeong Song, Joohyun Woo, HyunGoo Kim, Jun Woo Lee, Woosung Lim, Byung-In Moon, Kihwan Kwon

**Affiliations:** ^1^ Division of Cardiology, Department of Internal Medicine, Ewha Womans University College of Medicine, Ewha Womans University Mokdong Hospital, Seoul, Republic of Korea; ^2^ Department of Surgery, Ewha Womans University College of Medicine, Ewha Womans University Mokdong Hospital, Seoul, Republic of Korea

**Keywords:** cancer therapy-related cardiac dysfunction, cardiac extracorporeal shock wave therapy, left ventricular global longitudinal strain/LV GLS, breast cancer, doxorubicin

## Abstract

**Background:**

Doxorubicin is a highly effective anti-cancer drug that causes left ventricular (LV) dysfunction and induces late-onset cardiomyopathy. However, an effective and clinically applicable preventive treatment is yet to be discovered.

**Objective:**

Cardiac-Extracorporeal shockwave therapy (C-ESWT) has been suggested to treat inflammatory and ischemic diseases and protect cardiomyocytes from doxorubicin-induced cardiomyopathy. This study aims to assess the safety and efficacy of C-ESWT in the prevention of subclinical cardiotoxicity.

**Methods:**

We enrolled 64 breast cancer patients. C-ESWT group 33 patients were treated with our C-ESWT (200 shots/spot at 0.09 mJ/mm^2^ for 20 spots, 3 times every six weeks). The efficacy endpoints were the difference in left ventricular global longitudinal strain (LVGLS) change by 2D speckle tracking echocardiography and chemotherapy-related cardiac dysfunction (CTRCD). Echocardiography was performed on the baseline line and every 4 cycles of chemotherapy, followed by a follow-up 3,6 months after chemotherapy to compare the incidence of cardiomyopathy of subclinical LV dysfunction due to chemotherapy between the two groups.

**Results:**

Participants averaged 50 ± 9 years in age, 100% female. In the results of follow-up 6 months after the end of chemotherapy, there was a significant difference in delta LVGLS between the C-ESWT group and the control group (LVGLS; −1.1 ± 10.9% vs. −11.5 ± 11.6% *p*-value; <0.001). A total of 23% (15 patients) of patients developed CTRCD (Control group; 13 vs. C-ESWT group; (2). C-ESWT was performed safely without any serious adverse events.

**Conclusion:**

In this prospective study, C-ESWT established efficacy in preventing subclinical cardiotoxicity, especially in breast cancer patients using doxorubicin chemotherapy, and the safety of C-ESWT.

**Clinical Trial Registration:**

ClinicalTrials.gov, identifier (NCT05584163).

## Introduction

Doxorubicin is a well-known and widely used anthracycline class therapeutic agent for the treatment of breast cancer, blood cancer, and other types of cancer. Studies using neurohormonal antagonists drugs such as Angiotensin-converting enzyme (ACE) inhibitors or Angiotensin receptor blockers (ARB), beta-blockers, and others have been successful or unsuccessful in preventing doxorubicin-induced cardiomyopathy. Dexrazoxane, which was designated as an orphan drug by the FDA in 2014, is currently the only drug approved for the prevention of anthracycline-induced cardiotoxicity in children and adolescents aged 0–16 years (1, 2).

Currently, guidelines recommend the use of dexrazoxane when using Doxorubicin in children or when an adult has accumulated a certain threshold dose of Doxorubicin. While dexrazoxane is successful in suppressing anthracycline cardiotoxicity, it does not offer complete cardioprotection, since anthracyclines have several possible cardiotoxic mechanisms, and dexrazoxane only targets some of them (3–5). The cardiotoxicity associated with anthracycline use can range from subclinical cardiomyopathy to heart failure (HF) and even cardiac death. HF may occur within the first week of anthracycline treatment or it may take decades to develop (6). However, most cases occur within the first year after treatment (7).

Doxorubicin, an anthracycline used for clinical purposes, has been shown to induce oxidative stress and apoptosis of cardiomyocytes, limiting its long-term use due to cardiotoxicity (8, 9). This is supported by several studies which have demonstrated that doxorubicin increases the risk of cardiotoxicity with cardiac symptoms similar to those of dilated cardiomyopathy (10–12). Survivin, a member of the inhibitor of the apoptosis protein family, has been found to regulate cellular apoptosis and tumor progression in various cell types (13–15). As doxorubicin treatment induces cellular apoptosis in cardiomyocytes, survivin has been identified as a suitable therapeutic target for patients with DOX-induced cardiomyopathy. Therefore, upregulation of endogenous survivin levels may be a more reasonable potential therapy for doxorubicin-induced cardiomyopathy.

Extracorporeal shock wave (ESW) therapy has been used as a first-line treatment for stone diseases due to its high energy (16, 17). Low-energy ESW has been shown to have protective effects in various diseases associated with bones, tendons, and the musculoskeletal system, and to promote angiogenesis and improve cardiac injury after acute myocardial infarction (18). Previous studies have shown that low-energy ESW promotes angiogenic gene expression and activates the PI3K/Akt signaling pathway, which is involved in survivin expression (13, 19, 20, 21). Based on these findings, subsequent research has proposed ESW as a new specific and safe therapy against acute doxorubicin-induced cardiomyopathy, protecting cardiomyocytes by upregulating survivin in an *in vivo* model (22).

Therefore, this study aims to investigate whether Cardiac Extracorporeal Shock Wave Therapy (C-ESWT) can prevent subclinical cardiotoxicity [change of LVGLS and Cancer Therapy-Related Cardiac Dysfunction (CTRCD)], in patients receiving doxorubicin chemotherapy.

## Methods

Breast cancer patients receiving anthracycline-based chemotherapy were enrolled at Ewha Woman's Mokdong Hospital between June 2021 and December 2022, with the aim of recruiting at least 72 participants. Previous studies have shown a 2% difference in absolute LVGLS in the event of chemotherapy-induced cardiotoxicity. The expected mean of LVGLS in the treatment group is −20% with a standard deviation of 2.4, and the expected mean of −18.5% with a standard deviation of 2.4 in the untreated group is 66 patients with an error of 0.05 and power of 0.8, and we planned to enroll a total of 72 patients considering a 10% drop. Eligible participants had a normal cardiac function, as determined by screening echocardiography, and provided voluntary consent to participate. Participants were randomly assigned to either the treatment group or the control group. Inclusion criteria were patients aged 19 or older, with breast cancer scheduled to receive at least 3 cycles of anthracycline-based chemotherapy, and normal cardiac function. Exclusion criteria were patients with structural heart disease, intracardiac device presence, antiarrhythmic drugs that may affect the QT interval, and recent defibrillation due to AF or previous PCI or coronary artery bypass surgery. This study was registered at ClinicalTrials.gov, registration number NCT05584163.

64 patients were randomly divided into the ESWT group (*n* = 33) and control group (*n* = 31), with 8 patients excluded due to declining participation or inconvenient transportation. Patients in the ESWT group received ESWT and standard chemotherapy per guidelines, while those in the control group received standard chemotherapy containing doxorubicin.

### C-ESWT protocol

**Figure 1:**
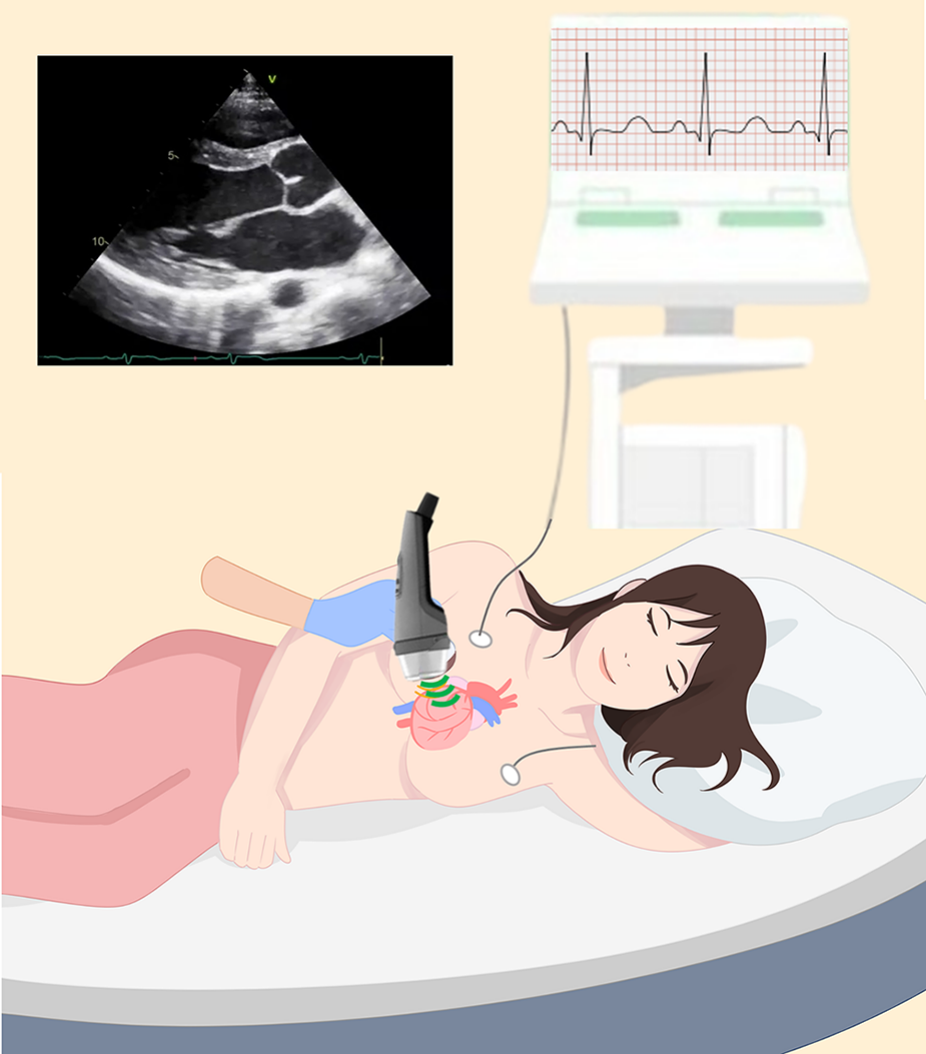
During the C-ESWT procedure. Illustration depicting C-ESWT procedure. The patient is lying down, the location of the myocardium is confirmed by echocardiography, a C-ESWT probe is applied, and the electrocardiogram is monitored. C- ESWT, Cardiac Extracorporeal Shock Wave Therapy.

One cycle of C-ESWT was performed according to the doxorubicin chemotherapy schedule: the doxorubicin chemotherapy schedule was every 3 weeks, and C-ESWT was performed every 2 chemotherapy cycles. Therefore, one cycle of C-ESWT was performed every 6 weeks. C-ESWT was performed three times per cycle, on the first, third, and fifth days of each cycle, and each patient received at least six C-ESWT sessions. The energy and the total number of C-ESWT therapy were set to 200 shots/spot at 0.09 mJ/mm^2^ for 40 spots per session. This protocol has been proven safe in previous studies targeting patients with coronary artery disease and refractory angina (23, 24). During the C-ESWT procedure, the location of the left ventricular myocardium was confirmed using echocardiography, and C-ESWT was performed. (Figure 1) The extracorporeal shock wave device used was the CENOWAVE model manufactured by HNT Medical, Inc. The extracorporeal shock wave device consists of a main unit that can control the intensity and frequency of shock waves and a shock wave head that delivers extracorporeal shock waves directly to the body parts. Shockwave heads are divided into multifocus type and focus type, and multifocus type was used in this study. 18.06 MPa pressure with 0.1EFD density energy was used in this study. The extracorporeal shock wave was performed under the supervision of a doctor by a medical practitioner who can acquire echocardiographic images to confirm the location of the myocardium, and the procedure took about 20 min. Additionally, an EKG was connected to monitor arrhythmia. At baseline and follow-up, peripheral venous blood samples were collected and analyzed for myocardial markers including creatine kinase (CK), creatine kinase phosphate-isozyme (CK-MB), and Troponin T (TnT). Additionally, the Complete Blood Count (CBC) and hepatorenal function indexes alanine aminotransferase (ALT), aspartate aminotransferase (AST), and lactic dehydrogenase (LDH) serum creatinine (Cr) were also measured.

### Echocardiography

Patients underwent echocardiography at baseline prior to starting anthracycline therapy, and again at 3 and 6 months after completing chemotherapy, which included doxorubicin. The echocardiograms were obtained using GE Ultrasound System and Philips Ultrasound Machines and included 3–5 cardiac cycles of 4-, 3-, and 2-chamber apical views. The images were digitally stored in raw format for later analysis. LVGLS was analyzed using a semiautomated speckle tracking technique (TOMTEC Imaging system GmbH Freisinger Strasse9, 85716 Unterschleissheim Germany) with a model of the entire left ventricle, including the 3 apical views. Inadequately tracked segments were excluded from the analysis. Two different observers analyzed the datasets and measured the left ventricular global longitudinal strain (LVGLS) using TOMTEC. Each observer analyzed the dataset twice, with the second review taking place 3 months after the first. The observers were blinded to each other during the analysis, and the second measurement of each observer was used to evaluate intraobserver reproducibility.

### Outcome

The primary outcome was the difference in LVGLS between baseline and the 3-month and 6-month follow-up in the ESWT group compared to the control group. The secondary outcome was the difference in the rate of CTRCD between the two groups. In this study, CTRCD was defined as a reduction in LVEF of >10 absolute percentage points to a value <50% as measured by echocardiography at any follow-up time point (25). These patients were defined as having definite CTRCD. Probable CTRCD was defined as a reduction in LVEF of >10 absolute percentage points to a value ≥50% accompanied by a relative reduction in LVGLS of >15%. Possible CTRCD was defined as a reduction in LVEF of <10 absolute percentage points to a value <50% or a relative reduction in GLS of ≥15% from baseline. This study defined patients meeting these criteria as having possible CTRCD (25).

### Statistical analysis

The statistical analysis was carried out using SPSS version 23.0 (SPSS Inc., USA). Continuous variables with a normal distribution were reported as mean ± standard deviation and analyzed using paired *t*-tests for baseline and follow-up comparison. Categorical variables were presented as frequency (*n*) or ratio and analyzed using the chi-square test. Rank data were analyzed using a non-parametric rank sum test. Intraobserver and interobserver reliabilities were measured using the intraclass correlation coefficient and the Bland-Altman analysis. A *p*-value of less than 0.05 (two-tailed) was considered statistically significant.

Informed consent was obtained from all participants and the study was approved by the IRB of Ewha Womans University Mokdong Hospital.

## Results

### Patient characteristics

**Figure 2:**
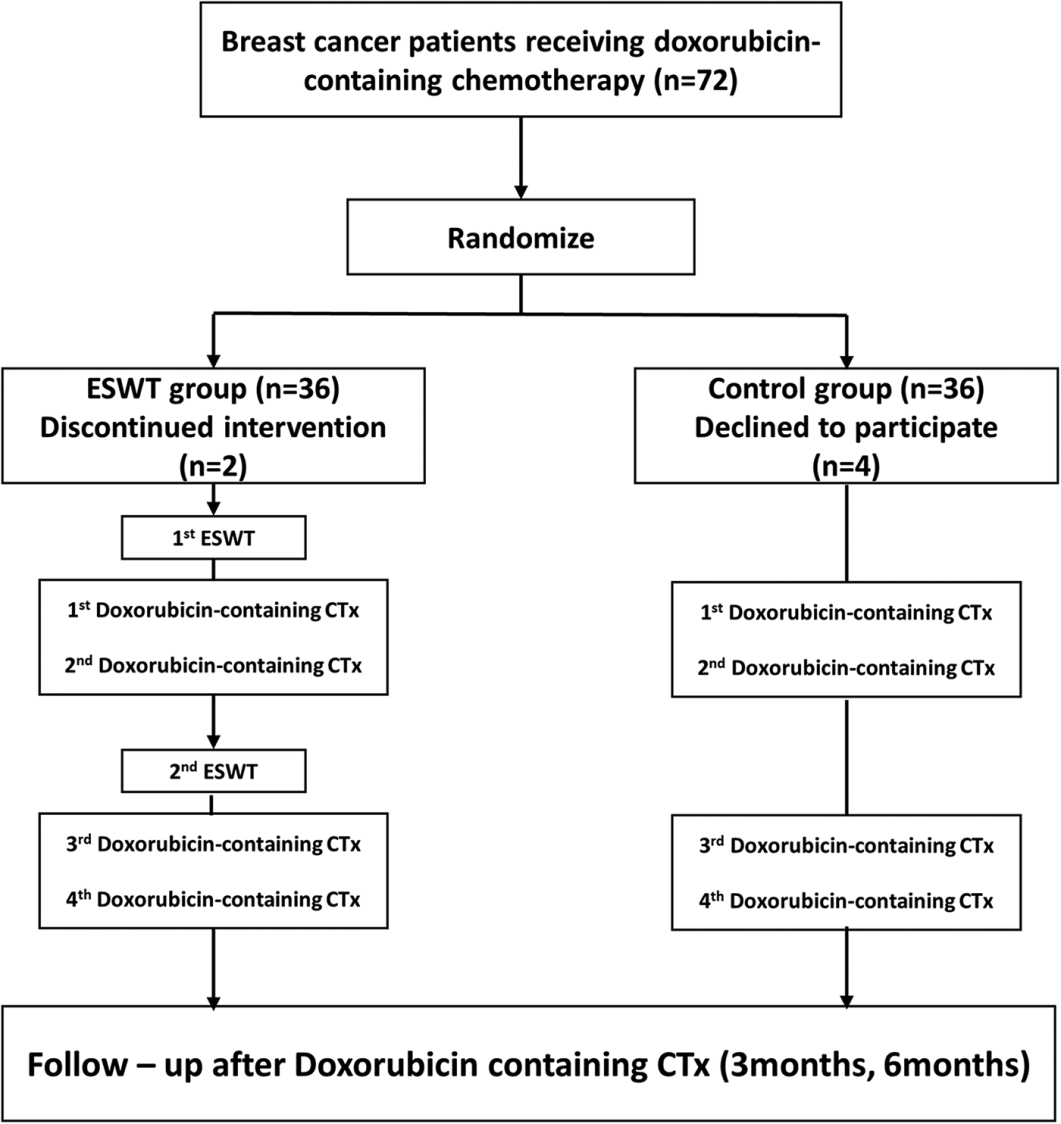
Prevention of CTRCD (doxorubicin-induced cardiomyopathy) protocol in the C-ESWT group and control group. CTRCD, Cancer Therapy-Related Cardiac Dysfunction, C- ESWT; Cardiac Extracorporeal Shock Wave Therapy.

Out of the 72 patients who were randomized, three patients (7%) did not receive follow-up (as shown in Figure 2). Five patients withdrew their consent or did not come back for imaging follow-up after the baseline study. The final analysis included 64 patients (33 in the ESWT treatment group and 31 in the control group). A comparison of the baseline clinical and cardiac imaging characteristics of the included patients between the two groups is presented in Table 1. All patients were women, with an average age of 50 ± 9 years (range 29 to 72 years). Common risk factors for heart failure were: 11 (17.2%) had hypertension, 4 (6.3%) had diabetes mellitus, 5 (7.8%) had dyslipidemia, and none were current or ex-smokers. The two groups had no significant difference in baseline EF or LVGLS. All patients had breast cancer and received anthracycline-based chemotherapy. The median doxorubicin equivalent dose was 241 mg/m^2^ [interquartile range (IQR): 238 to 245 mg/m^2^], and there was no significant difference between the C-ESWT and control groups (C-ESWT group: 241 mg/m^2^ [IQR: 237–244 mg/m^2^]; control group: 241 mg/m^2^ [IQR: 238–245 mg/m^2^]; *p* = 0.14).

**Table 1:** Patient characteristics.

	ESWT group (*N* = 33)	Control group (*N* = 31)	*P* value
Demographics			
Age, year	51 ± 7	50 ± 11	0.672
Female	34 (100)	32 (100)	–
Risk factors			
Diabetes	4 (11.8)	1 (3.1)	0.190
Hypertension	7 (20.6)	5 (15.6)	0.608
Dyslipidemia	4 (11.8)	2 (6.3)	0.440
Smoking	0	0	–
BMI, kg/m^2^	23.7 ± 5.0	22.7 ± 4.0	0.363
Previous cardiovascular disease	0	0	–
Baseline medical therapy			
Beta-blocker	0	1 (3.1)	0.306
ACEI or ARB	6 (17.6)	2 (6.3)	0.161
Statin	4 (11.8)	2 (6.3)	0.440
Observations			
Systolic blood pressure, mmHg	121 ± 15	119 ± 15	0.539
Diastolic blood pressure, mmHg	73 ± 11	73 ± 10	0.974
Heart rate, beats/min	73 ± 11	73 ± 14	0.941
Chemotherapy			0.112
Neoadjuvant	5 (14.7)	10 (31.3)	
Adjuvant	29 (85.3)	22 (68.8)	
Additional therapy after doxorubicin			0.647
Paclitaxel	30 (88.2)	28 (87.5)	
Docetaxel	3 (8.8)	1 (3.1)	
Trastuzumab	0	2 (6.3)	
Letrozole	1 (2.9)	1 (3.1)	
Cumulative doxorubicin dose, mg/m^2^	240 ± 6	243 ± 10	0.133
EF			
Baseline, %	65 ± 5	66 ± 4	0.366
GLS			
Baseline, %	−20.9 ± 2.6	−20.7 ± 2.2	0.751
Baseline <-18%	31 (90.9)	29 (90.6)	
Baseline −16% to −18%	3 (9.1)	3 (9.4)	
Baseline >-16%	0	0	
Laboratory			
Baseline CK	54 ± 25	68 ± 33	0.366
Baseline CK-MB	1.3 ± 1.2	1.0 ± 0.4	0.610
Baseline Troponin T	0.01 ± 0.011	0.02 ± 0.014	0.384

### Primary outcome—LVGLS

**Figure 3:**
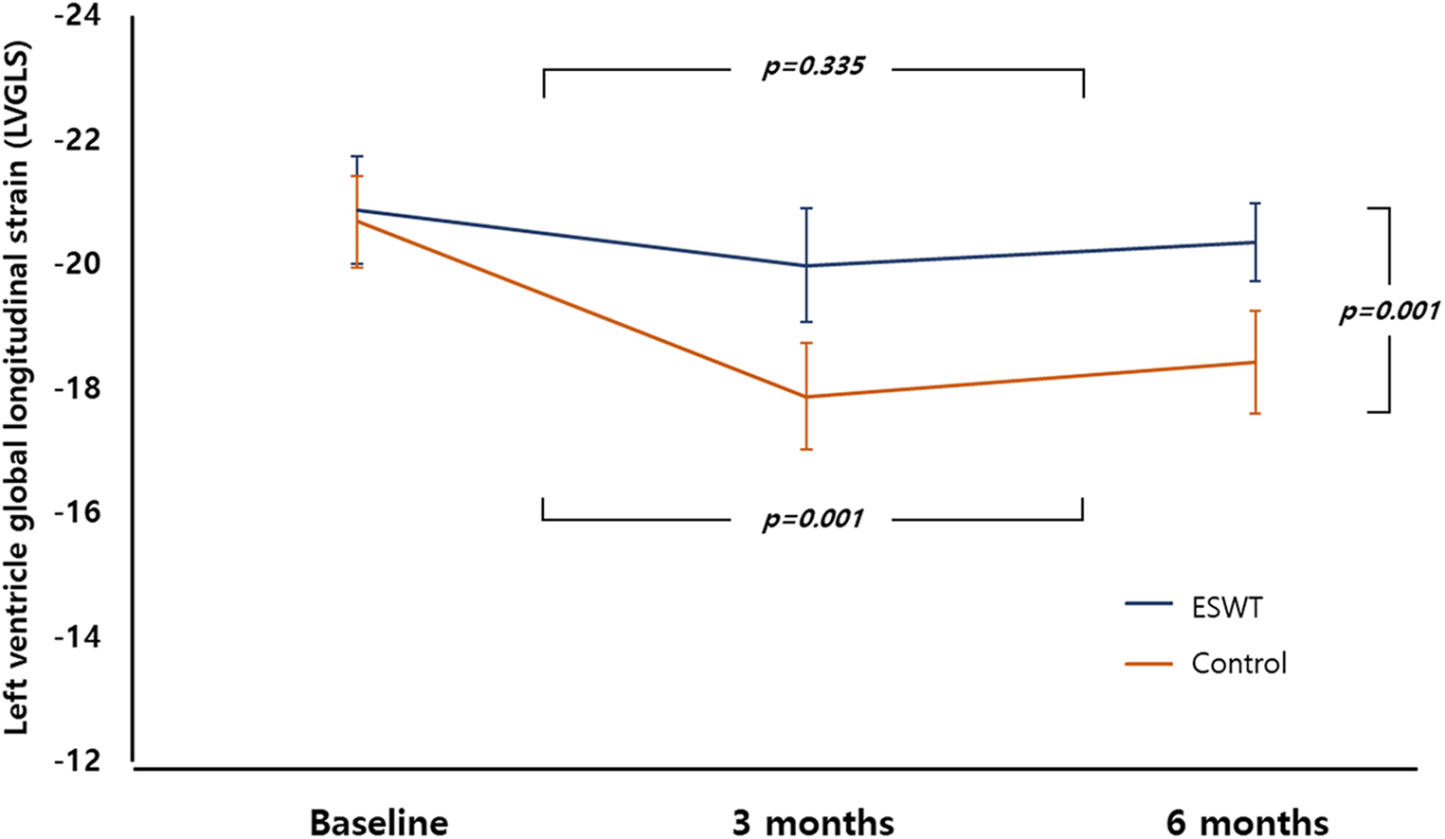
Trend of LVGLS at baseline, 3 months, 6 months between the two groups. LVGLS, left ventricular longitudinal global strain.

There was no significant difference in baseline LVGLS between the two groups (C-ESWT −21.4% ± 2.9% vs. control group −20.7% ± 2.5%, *p*-value 0.351). However, at 3 months after anthracycline-based chemotherapy, including doxorubicin, the GLS values were −20.9% ± 2.9% and −17.9% ± 2.7% for the C-ESWT and control groups, respectively. These values showed a statistically significant difference in the change from baseline, with −1.6% vs. −13.5%, respectively. A similar trend was observed at 6 months, where the GLS values also showed statistically significant differences between the two groups and from baseline (Table 2, Figure 3). However, there was no significant difference in LVEF, LVEDVi, and LVESVi between the two groups at 3 and 6 months (Table 2).

**Table 2:** EF and LVGLS measures throughout doxorubicin therapy.

	ESWT group (*N* = 33)	Control group (*N* = 31)	*P* value	Mean difference
LVEF, %				
Baseline	64.8 ± 4.9	66.3 ± 4.3	0.213	
Post—3 months	64.7 ± 4.4	62.5 ± 7.4	0.150	
Change from baseline (%)	0.0 ± 5.7	−5.7 ± 10.1	0.008	
Post—6 months	64.7 ± 4.4	63.5 ± 5.9	0.413	
Change from baseline (%)	−0.2 ± 7.7	−3.7 ± 7.3	0.076	
LVEDVi, ml/m^2^				
Baseline	42.5 ± 8.7	44.7 ± 10.9	0.371	
Post—3 months	43.1 ± 9.2	43.3 ± 10.7	0.942	
Change from baseline (%)	2.9 ± 18.9	−0.9 ± 21.8	0.458	
Post—6 months	43.9 ± 10.2	41.4 ± 9.3	0.359	
Change from baseline (%)	5.3 ± 21.5	−5.7 ± 20.4	0.067	
LVESVi, ml/m^2^				
Baseline	17.6 ± 5.3	19.2 ± 6.2	0.269	
Post—3 months	18.6 ± 4.8	21.6 ± 6.4	0.036	
Change from baseline (%)	11.4 ± 34.5	16.9 ± 30.2	0.501	
Post—6 months	18.4 ± 5.3	20.5 ± 5.3	0.161	
Change from baseline (%)	13.0 ± 41.4	10.5 ± 25.5	0.795	
GLS, %				
Baseline	−21.4 ± 2.9	−20.7 ± 2.5	0.351	−0.64 (−2.00, 0.72)
Post—3 months	−20.9 ± 2.9	−17.9 ± 2.7	0.002	−3.05 (−4.46, −1.63)
Change from baseline (%)	−1.6 ± 10.1	−13.5 ± 10.5	<0.001	11.9 (6.74, 17.06)
Post—6 months	−21.0 ± 2.5	−18.4 ± 2.7	<0.001	−2.53 (−3.89, −1.16)
Change from baseline (%)	−1.1 ± 10.9	−11.5 ± 11.6	<0.001	10.4 (4.50, 16.36)

### Secondary outcome—CTRCD

There were no significant differences in LVEF or GLS between the two groups at baseline, as shown in Table 1. Cardiotoxicity occurring at any time during the study was observed only in two patients in the control group. After completion of anthracycline-based chemotherapy, probable CTRCD was not observed in either group. But possible CTRCD occurred in two patients in the C-ESWT group at 6 months and in 11 patients in the control group, demonstrating statistical significance between the two groups. The occurrence of any type of CTRCD during follow-up was significantly higher in the control group than in the C-ESWT group (13 events in control group vs. 2 events in C-ESWT group; *p*-value = 0.001) as shown in Table 3.

**Table 3:** Cancer therapy-related cardiac dysfunction (CTRCD) between ESWT and control group.

	ESWT group (*N* = 33)	Control group (*N* = 31)	*P* value
Total	2	13	0.001
Cardiotoxicity	0	2	0.143
Probable subclinical cardiotoxicity	0	0	–
Possible subclinical cardiotoxicity	2	11	0.003

### Safety

All patients tolerated ESWT well without any major complications. Only 3 patients (9%) reported pain around the C-ESWT site. However, no bruises or skin lesions were observed at the site where extracorporeal shock wave therapy was performed. Throughout the trial, there were no clinically significant increases in BNP, CK, CK-MB, or troponin I level in individual patients. The mean BNP levels at screening, 3-month, and 6-month follow-up were 172.8 ± 286.9, 147.9 ± 303.8, and 173.7 ± 299.1 pg/ml, respectively, and there were no significant changes in EKGs from baseline to follow-up. In addition, EKG monitoring was performed during the C-ESWT and no significant arrhythmias occurred.

## Discussion

This pilot study proposes that C-ESWT therapy serves as a secure, non-invasive, and effective preventative measure for doxorubicin-induced cardiomyopathy in breast cancer patients, where no established preventive drug or method currently exists. The C-ESWT group exhibited a significantly lower incidence of CTRCD, with no observed serious adverse events related to extracorporeal shock wave therapy.

Doxorubicin is renowned for inducing oxidative stress and cardiomyocyte apoptosis, leading to cardiotoxicity. A previous article suggested survivin, an apoptosis inhibitor protein, as a promising target for treating DOX-induced cardiomyopathy due to its regulation of cellular apoptosis. ESWT usage promotes an increase in survivin, and its cardioprotective effect has been demonstrated *in vivo* studies. This study is pivotal in confirming the safety and myocardial protective effect of ESWT through LVGLS analysis in humans.

Previous studies utilizing ESWT on the heart primarily aimed at improving symptoms or left ventricular systolic function (LVEF) in refractory angina patients (23, 24, 26). This study stands out as the first to explore whether C-ESWT can prevent doxorubicin-induced cardiomyopathy in a homogeneous group of female breast cancer patients. Despite doxorubicin's effectiveness in treating breast cancer, sarcoma, and hematologic malignancies, its use is restricted due to its toxicity, particularly cardiac toxicity. Ongoing efforts to investigate the efficacy of neurohormonal antagonists, such as renin-angiotensin-aldosterone inhibitors and beta-blockers, in reducing cardiotoxicity have not been universally adopted in clinical practice due to inconsistent results (27–32). Guidelines and the US FDA do not offer indications for using dexrazoxane to prevent cardiac toxicity in adult patients using non-high doses of doxorubicin or from the outset. Consequently, there are currently no guidelines-based recommendations for a preventive method to lower the risk of cardiomyopathy associated with low-dose doxorubicin use (33).

The definition criteria for possible and probable cardiomyopathy outlined in recent guidelines incorporate LVGLS. LVGLS is recognized for its ability to predict future EF reduction when it significantly decreases before EF reduction in chemotherapy patients (34). Widely utilized, LVGLS is strongly correlated with prognosis not only in CTRCD but also in heart failure, valvular disease, and myocardial disease. While LVGLS does not meet the definition of definite CMP in CTCRD, it exhibits a better or potential earlier diagnosis of subclinical LV dysfunction. Therefore, ESWT has proven to be a valuable tool in preventing possible and probable cardiomyopathy in these two groups. If long-term follow-up results confirm this, it may serve as robust evidence for preventing CTRCD using C-ESWT.

Prior studies indicated an increased frequency of doxorubicin-induced cardiotoxicity with a higher cumulative dose (35). However, even in patients not undergoing high-dose doxorubicin chemotherapy, a significant incidence of probable cardiomyopathy is observed according to the CTRCD criteria (36). This underscores the necessity for preventive measures to avert cardiotoxicity from the outset, not only in high-dose doxorubicin-based chemotherapy (as recommended by guidelines allowing dexrazoxane use) but also in cases of low-dose doxorubicin-based chemotherapy in adult cancer patients.

To ensure safety during shock wave therapy, continuous EKG monitoring was implemented to detect arrhythmias, and no new cases of arrhythmia were observed in subsequent follow-up EKGs. Additionally, serial CBCs performed during chemotherapy did not reveal any patients with suspected hemolytic anemia, and no serious adverse events occurred.

### Study strengths and limitations

To our knowledge, our study represents the first randomized controlled investigation into the preventive effects of C-ESWT on CTRCD in patients undergoing anthracycline-based cancer therapy. A significant strength of our study lies in the implementation of highly standardized entry criteria, resulting in a relatively homogeneous patient population despite the small sample size. Additionally, our focus on patients with relatively low heart failure risk factors enhances the robustness of our findings. To mitigate differences in LVGLS between vendors, all images were anonymized and analyzed using the TOMTEC program. However, limitation of this study is inter-vendor variability in interpreting the results. Although the vendor-independent 2D strain software provided moderate correlations between the LVGLS values of the ultrasound images obtained from the same subjects using different vendors, LVGLS measurements should preferably be interpreted relative to previous examinations with the same machine and software or vs. vendor-specific reference values (36–38). In this study, images were acquired using GE and Philips equipment, and LVGLS values were analyzed by TOMTEC. The difference in reference values between vendors should be considered when viewing the results. The analysis was conducted twice by two investigators, and the study's strengths include confirming the LVGLS trend at 3 and 6 months post-completion of anticancer therapy. As well-established in the literature, our study also demonstrated a high level of agreement in LVGLS measurements both between and within examiners (Supplementary Figure S1).

While our data suggest limited adverse events associated with C-ESWT therapy, caution is warranted in interpreting these results due to the small sample size. Larger multicenter confirmatory studies, incorporating sham-controlled randomized trials, are imperative to establish the beneficial effect of ESWT in patients undergoing anthracycline-based cancer therapy.

Despite the limited participant number, available data indicate that C-ESWT has few adverse events. However, interpreting the present data requires caution for a more accurate understanding of the potential benefits of this technique, necessitating larger standardized studies. Therefore, larger multicenter confirmatory studies, including randomized trials with sham control, are necessary to validate the beneficial effects of C-ESWT in patients undergoing anthracycline-based cancer therapy.

Furthermore, although no statistically significant difference in definite cardiomyopathy was observed between the two groups, it exclusively occurred in the control group. The incidence of definite cardiomyopathy in the control group did not significantly differ from other studies, and the occurrences of probable and possible cardiomyopathy were not excessively high compared to some previous research (39, 40). However, this is constrained by the small overall sample size. The absence of definite cardiomyopathy in the C-ESWT group aligns with prior studies demonstrating the effectiveness of C-ESWT in treating acute doxorubicin-induced cardiomyopathy (22).

## Conclusion

In conclusion, this pilot study demonstrates that Cardiac Extracorporeal Shock Wave Therapy is a safe and effective method for preventing CTRCD especially doxorubicin-induced cardiomyopathy in breast cancer patients. The safety of C-ESWT therapy was confirmed, as no serious adverse events were observed. These findings have important implications for the prevention of CTRCD and the potential use of C-ESWT therapy as a preventive measure.

## Data Availability

The raw data supporting the conclusions of this article will be made available by the authors, without undue reservation.
