# Supplementary figures and images for: A prospective randomized controlled trial to determine the safety and efficacy of extracorporeal shock waves therapy for primary prevention of subclinical cardiotoxicity in breast cancer patients without a cardiovascular risk treated with doxorubicin
Source: Front Cardiovasc Med. 2024 Feb 7;11:1324203. doi: 10.3389/fcvm.2024.1324203 (PMC10879594; doi:10.3389/fcvm.2024.1324203)

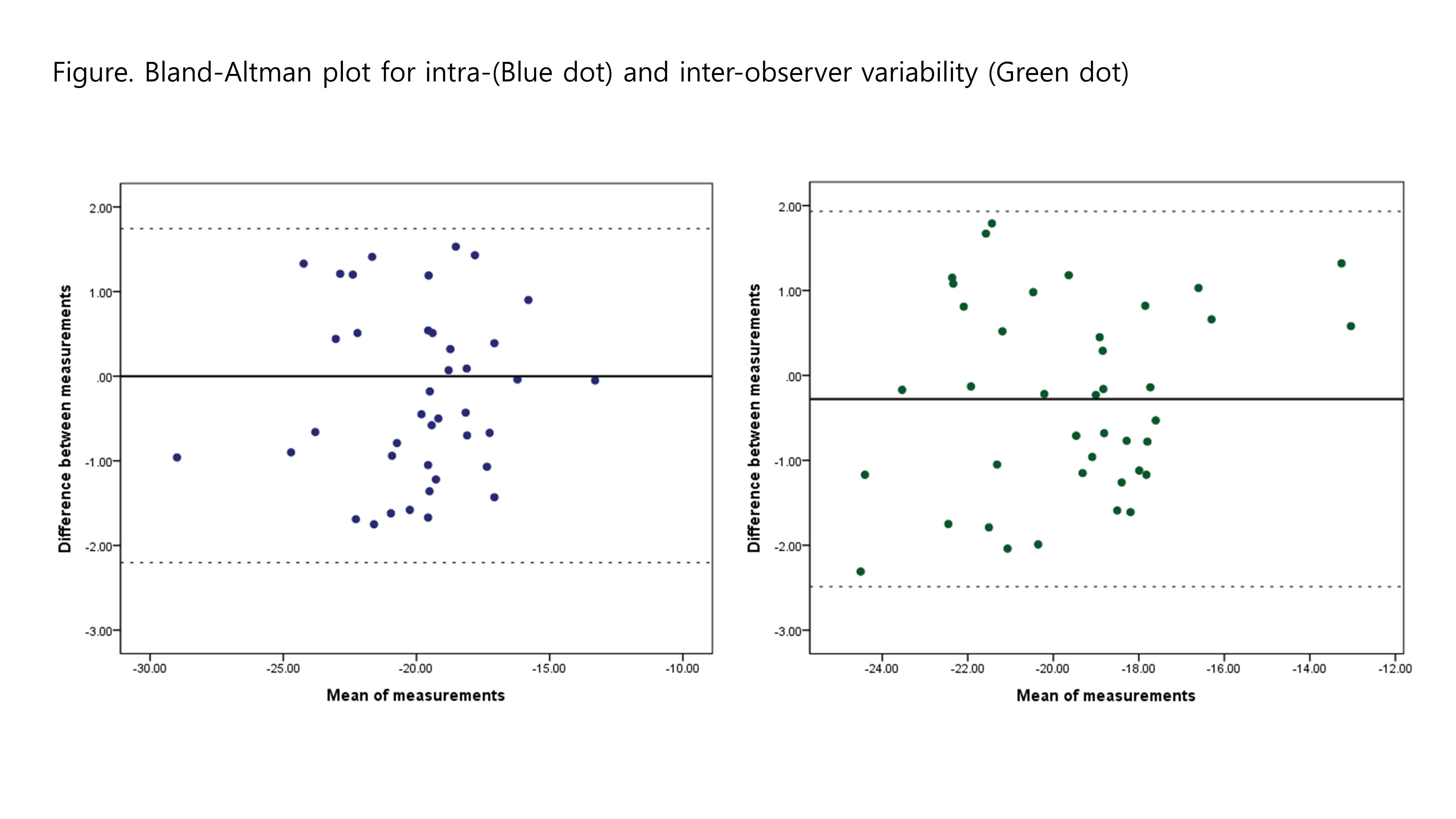

Supplement: Supplementary file 1 [file Image1.tif]
